# A dataset representing the impact of cyanide on the fatty acid profile of *Scenedesmus obliquus*

**DOI:** 10.1016/j.dib.2019.103900

**Published:** 2019-04-04

**Authors:** Lukhanyo Mekuto, Dakalo Musingadi

**Affiliations:** Department of Chemical Engineering, University of Johannesburg, Johannesburg, 2028, South Africa

**Keywords:** Biodegradation, Free cyanide, *Scenedesmus obliquus*, Fatty acids

## Abstract

The data presented represents the biodetoxification of free cyanide (CN^−^) by *Scenedesmus obliquus*and the subsequent fatty acid profile of the tested algal specie. This algal organism can use cyanide as a source of carbon and/or nitrogen for its growth. The organism was able to degrade 100 and 150 mg CN^−^/L to 3.7 and 12.4 mg CN^−^/L respectively, after 192 h. The main fatty acids which were detected were fatty acids with C_16_—C_18_ and were observed to be 97.7% and 99.55% from cultures with 100 and 150 mg CN^−^/L respectively. High CN^−^ concentrations proved to be favourable for the accumulation of polyunsaturated fatty acids (≥75%), thus demonstrating the biofuel production capacity of microalgal species in bioremediation of hazardous substances.

**Table:** Specification table

Subject area	Energy and Environmental Biotechnology
More specific subject area	Biofuels and Bioremediation
Type of data	Tables
How data was acquired	Direct transesterification followed by GCxGC-TOF-MS (Leco Corporation St. Joseph, MI, USA) analysis of the fatty acids
Data format	Analysed
Experimental factors	Microalgae were tested for CN^−^ biodegradation at different concentrations and the biomass was harvested after the experimental run for direct transesterification [1].
Experimental features	The fatty acids were analysed using GCxGC-TOF-MS using the Restek Rtx-5siLMS (30 m × 250 μm × 0.25 μm) as the first dimension column and Restek Rxi17siLMS (1 m × 250 μm × 0.25 μmd.f.) as a second dimension column.
Data source and location	Johannesburg, South Africa (26.2041° S, 28.0473° E) University of Johannesburg (26.1945° S, 28.0576° E)
Data accessibility	Data is provided in the article
Related research article	The article related to this article is still under construction.

**Table:** 

**Value of the data** • This data demonstrated the ability of *Scenedesmus obliquus* to degrade high concentrations of free cyanide at alkaline conditions. • The fatty acid analysis revealed an accumulation of polyunsaturated fatty acids with an increase in the initial concentration of free cyanide. • This data can be used as a motivation for the utilization of microalgal species for the treatment of environmentally hazardous compounds while recovering fatty acids for subsequent bioenergy production.

## 1 Data

The data presented herein shows the impact that free cyanide has on the fatty acid profile of a cyanide-degrading microalgae. Table 1 demonstrates the summarised growth and degradation data while Table 2 demonstrates the fatty acid profiles of S*cenedesmus obliquus*.

**Table 1:** Maximum specific growth rate (μ_max_), minimum doubling time (T_d min_), biomass concentration (X_max_), CN^−^ degradation rate and efficiency measured over 192 h batch growth cycle.

Parameters	Control	100 mg CN^−^/L	150 mg CN^−^/L
μ_max_ (days^−1^)	0.81 ± 0.16	0.34 ± 0.09	0.16 ± 0.07
T_d min_ (days)	0.42	0.11	0.06
X_max_ (g.L^−1^)	1.71 ± 0.23	0.85 ± 0.11	0.68 ± 0.12
Degradation rate (mg.L^−1^. day^−1^)	–	0.50	0.72
Degradation efficiency (%)	–	99.97	91.67

**Table 2:** Main fatty acids of *S. obliquus* under different CN^−^ concentrations.

Fatty acid	Content (%)		
	Control	100 mg CN^−^/L	150 mg CN^−^/L
C14:0	0.37	0.18	0.06
C15:0	0.17	n.d.	0.02
C16:0	32.4	28.30	22.17
C16:1	0.10	0.07	0.11
C16:2	3.00	2.21	3.11
C16:3	5.36	5.00	4.32
C17:0	0.31	0.10	n.d
C18:0	2.01	2.03	2.23
C18:1	0.55	0.93	1.87
C18:2	9.12	15.30	16.42
C18:3	44.52	43.76	49.32
C20:0	0.13	0.09	n.d.
C20:1	0.11	0.27	n.d.
C20:5	1.01	1.53	0.06
Saturated	35.39	30.7	24.48
Unsaturated	36.77	69.07	75.21
C_16_—C_18_	97.35	97.70	99.55

n.d.: undetectable.

Fatty acids are abbreviated with a number before the colon shows the number of carbon atoms number while the number after the colon signifies the number of double bonds.

## 2 Experimental design, materials and methods

### 2.1 Microorganism and experimental conditions

*Scenedesmus obliquus* was obtained from the culture collection of the Department of Biotechnology, University of Johannesburg, South Africa. The culture was maintained in 3 N Bold Basal Medium (3-fold Nitrogen) at a pH of 9.5 in a bubble photobioreactor constructed of Plexiglas with 40 cm height, 10 cm external diameter and a 3 L working volume. Air enriched with 0.05% v/v CO_2_ was sparged at 2 L. min^−1^ and light (8900 lx) was provided by two 18 W fluorescent bulbs (Osram). The nitrogenous compound in 3 BBM medium was replaced by the 50 mg CN^−^/L and this culture was enriched for a period of 45 days prior to commencement of the experimental run. This was done to adapt the culture to CN^−^. The experiment setup was the same as described previously with minor modifications; the nitrogenous compound in the 3 BBM medium was replaced with free cyanide at concentrations of 100 and 150 mg CN^−^/L. After the experimental run, which was conducted over a 192 h period, the algal biomass was recovered by centrifugation (12,000 rpm for 10 min at 4 °C), followed by fatty acid analysis.

### 2.2 Biomass quantification and fatty acid analysis

A standard curve between cell dry weight and optical density was constructed by measuring cell density at 750 nm once a day while cell dry weights were measured using the procedure developed by Ref. [1]. The fatty acid content and profile was measured using direct transesterification and gas chromatography [2]. The maximum specific growth rate, minimum doubling time and the maximum biomass concentration was calculated according to the procedure described by Ref. [2]. The degradation rate and efficiency was determined as described by Ref. [3].
