# Supplementary material for: A dataset representing the impact of cyanide on the fatty acid profile of Scenedesmus obliquus
Source: Data Brief. 2019 Apr 4;24:103900. doi: 10.1016/j.dib.2019.103900 (PMC6463215; doi:10.1016/j.dib.2019.103900)
Supplement: Multimedia component 1 [file mmc1.pdf]

Conflict of interest statement

A dataset representing the impact of cyanide on the Fatty acid profile of *Scedesmus obliquus*

Dear Editor

The authors of the aforementioned manuscript declare that there is no conflict of interest that is associated with the submitted manuscript. That the manuscript has not been submitted to other journals for possible publications while it is submitted in *Data in Brief*. The corresponding author would also like to bring to the attention of the editorial team that the second author in this manuscript is deceased, hence he cannot sign.

Lukhanyo Mekuto

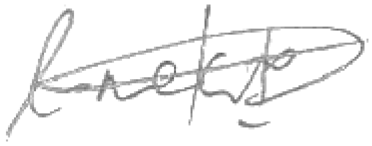

Dakalo Musingadi
